# The Conserved GTPase LepA May Contribute to the Final Proper Stabilization of the 3′ Domain of the 30S Subunit During Ribosome Assembly

**DOI:** 10.3390/ijms27010489

**Published:** 2026-01-03

**Authors:** Olesya Kravchenko, Elena Maksimova, Timur Baymukhametov, Irina Eliseeva, Elena Stolboushkina

**Affiliations:** 1 Institute of Protein Research, Russian Academy of Sciences, 142290 Pushchino, Russia; olesyak@vega.protres.ru (O.K.); maksimova.em@vega.protres.ru (E.M.); yeliseeva@vega.protres.ru (I.E.); 2 National Research Center “Kurchatov Institute”, Akademika Kurchatova pl. 1, 123182 Moscow, Russia; baymukhametov.timur@gmail.com

**Keywords:** ribosome assembly, cryo-EM, 30S subunit maturation, LepA

## Abstract

The function of the highly conserved GTPase LepA, a homolog of elongation factor EF-G, remains unknown in translation. However, there is biochemical data that it implicates in the 30S ribosomal subunit biogenesis. Here, using cryo-electron microscopy, we characterized 30S subunits isolated from an *Escherichia coli* strain with a deleted *lepA* gene. The cryo-EM maps for ∆*lepA* 30S particles were divided into classes corresponding to consecutive assembly intermediates: from particles characterized by unformed helices h44/h45 of the central decoding center (CDR) and highly flexible head, through intermediates with a distorted CDR and a partial stabilization of the head, to near-mature 30S subunits with correctly docked h44 in the CDR, accessible 3′ end of 16S rRNA for translation but significant flexibility in head domain. Cryo-EM analysis of Δ*lepA* 30S intermediates revealed that they predominantly proceed to nearly mature functional state and exhibit suboptimal flexibility in the head domain. This finding suggests that LepA likely contributes to the final proper stabilization of the 3′ domain of the 30S subunit during ribosome assembly.

## 1. Introduction

The function of LepA, a highly conserved translational GTPase, is currently unknown. Despite its wide phylogenetic distribution in bacteria (present in the cytosol), archaea, and eukaryotes (in mitochondria; in plants, and also in chloroplasts), LepA is non-essential for cellular viability. The deletion of the *lepA* gene has pleiotropic effects across species. In bacteria, the absence of LepA impairs cell growth under various stress conditions, including low pH media, extreme temperatures, high magnesium levels [1], and the presence of potassium tellurite [2] or specific acids [3]. Furthermore, in *Streptomyces coelicolor*, the *lepA* deletion leads to antibiotic overproduction [4]. In mycobacteria, the absence of LepA reduces the amount of membrane porins, leading to drug tolerance phenotypes such as rifampicin resistance [5,6]. In eukaryotes, the loss of LepA results in severe structural and functional defects in mitochondria, specifically impairing the assembly of the electron transport chain [7]. In mice, for instance, *lepA* knockout causes male infertility due to mitochondrial abnormalities in spermatocytes [8]. Similarly, in plants, the absence of the chloroplast-localized LepA homolog inhibits photosynthetic activity [9].

LepA is a paralog of EF-G, which promotes tRNAs translocation on the ribosome during the elongation cycle [10]. LepA consists of six domains, four of which are shared with EF-G [11]. In contrast to EF-G, which possesses a domain IV that acts as a “door-stop” to prevent reverse sliding of mRNA-tRNA, the protein LepA has a reduced EF-G’s domain IV and a unique C-terminal domain (CTD). This CTD extends into the peptidyl transferase center to interact with A- and P-site tRNAs. The combination of this structural similarity to EF-G and the absence of a full-length EF-G’s domain IV led to the hypothesis that LepA functions as a back-translocase [12].

The proposal that LepA catalyzes reverse translocation originated from a toe printing assay that prompted its renaming to elongation factor 4 (EF4) [12]. However, subsequent investigations failed to reproduce this effect. Kinetic analyses using fluorescently labeled reporters (mRNA and tRNAs) demonstrated that the mRNA-tRNA reverse movement in the presence of LepA occurred at a rate similar to that of spontaneous back-translocation [13], and an independent reassessment of the toe printing technique detected no this activity of LepA [14]. Further kinetic studies revealed that LepA could bind both pre- (PRE) and post-translocation (POST) ribosomal states, with a preference for the pre-translocation PRE complex [15]. Structural studies indicate that LepA binding remodels the A-site tRNA, displacing its acceptor stem from the peptidyl transferase center [16]. The functional significance of this distortion of the A-site tRNA remains unknown.

To investigate the physiological role of LepA, Balakrishnan et al. screened for synthetic genetic interactions of the Δ*lepA* mutation in *E. coli* [14]. They identified double mutants of Δ*lepA* with deletions in *dksA*, *molR1*, *tatB*, *tonB*, *tolR*, *ubiF*, *ubiG*, *ubiH* or *rsgA* exhibiting a significantly reduced growth rate. These genes are involved in distinct cellular processes, including gene regulation (*dksA*, *molR1*), respiration (*ubiF*, *ubiG*, *ubiH*), transport (*tatB*, *tonB*, *tolR*), and ribosome assembly (*rsgA*). This spectrum of genetic links suggests that LepA contributes, either directly or indirectly, to these processes.

Subsequent work by Gibbs et al. has implicated LepA in the 30S ribosomal subunit biogenesis [17]. The deletion of *lepA* leads to the accumulation of immature 30S particles, which are characterized by the presence of 17S rRNA precursor and an underrepresentation of specific late assembly proteins S3, S10, S14. These proteins are critical for the proper folding of the 3′ domain of the 16S rRNA. Furthermore, Gibbs et al. also established a genetic interaction between *lepA* and *rsgA*, which encodes a ribosome-dependent GTPase involved in small subunit biogenesis [18,19,20]. The ∆*lepA* mutation significantly exacerbated the growth defect of the ∆*rsgA* strain, increasing the *E. coli* doubling time. Taken together, these data indicate that LepA may participate in the assembly of the 30S ribosome subunit.

Here, we report cryo-EM structures of 30S assembly intermediates accumulated in an *lepA* null *E. coli* strain. The deletion of *lepA* caused distortions in the 3′ domains: the head (major domain) and helix 44 (h) with h45 (minor domain). These helices are essential components of the central decoding center. The cryo-EM maps for ∆*lepA* 30S particles were divided into classes corresponding to consecutive assembly intermediates: from particles characterized by unformed helices h44/h45 and highly flexible head, through intermediates with a distorted CDR and a partial stabilization of the head, to near-mature 30S subunits with correctly docked h44 in the CDR, accessible 3′ end of 16S rRNA for translation but significant flexibility in head domain. Cryo-EM analysis of Δ*lepA* 30S intermediates revealed that they predominantly proceed to nearly mature functional state and exhibit suboptimal flexibility in the head domain. This finding suggests that LepA likely contributes to the final proper stabilization of the 3′ domain of the 30S subunit during ribosome assembly.

## 2. Results

### 2.1. Characterization of the lepA Null Strain of E. coli

**Figure 1:**
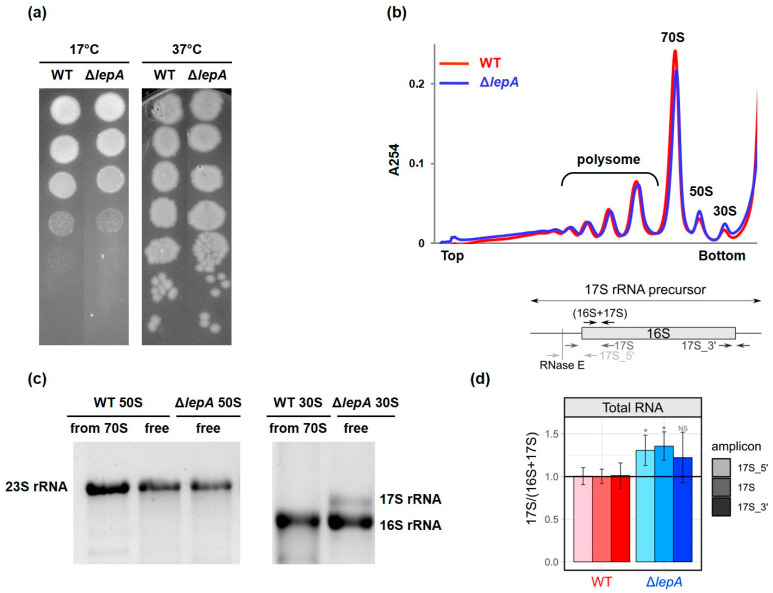
Comparison of wild-type (WT) and *ΔlepA* strains of *E. coli*. (**a**) Bacterial growth on LB-agar plates at 37 and 17 °C (consecutive dilutions in 1:10 steps). (**b**) Sucrose density gradient fractionation of the lysates of wild-type and *ΔlepA* cells collected at exponential growth phase. (**c**) Electrophoretic analysis of rRNA from small and large ribosomal subunits purified from 30S, 50S and 70S fractions of WT and Δ*lepA* cell lysate preparations. (**d**) Accumulation of 17S rRNA precursor in the wild-type and *ΔlepA* strains of *E. coli*. The bar plot shows the ratio of 17S/(16S + 17S), quantified by RT-qPCR. Y-axis: the relative ratio calculated as ΔΔCt and presented as fold change against the mean values for the wild-type (WT) strain. Whiskers: ± SD, * *p* < 0.05, NS = not significant, two-tailed Student’s *t*-test. A schematic of the 17S rRNA precursor illustrates the binding positions of the primers used for amplification. Diagram of the 17S rRNA precursor and corresponding amplicons on the right. The 5′ to 3′ orientation is indicated by the color gradient (light to dark).

The *E. coli* Δ*lepA* strain from the Keio collection [21] was used as the source of immature 30S subunits. This strain displayed a slight but obvious cold-sensitive phenotype (slow bacterial growth at low temperatures), consistent with a previous data (Figure 1a) [1]. However, we did not observe any significant changes in the ribosome profile (Figure 1b). The levels of free subunits, 70S ribosomes and polysomes were similar to those of the wild-type strain, differing from those reported by Balakrishnan et al. [14]. This distinction could be explained by the use of strains with diverse genotypic backgrounds in our work and that of Balakrishnan. Nevertheless, our Δ*lepA* strain (JW2553) accumulated the 17S rRNA precursor. The RNA gel analysis revealed that free 30S subunits contain 17S rRNA, indicating that these subunits are immature particles (Figure 1c). Using quantitative real-time PCR (qPCR) with primers specific to the precursor sequences (Figure 1d), we estimated that the levels of 17S rRNA precursor were 20% to 30% higher in the Δ*lepA* mutant than in the wild-type strain. This result is in good agreement with previous data [17]. Immature 30S subunits were purified from Δ*lepA* strain following our protocol developed for Δ*rbfA* 30S subunit purification [22].

### 2.2. LepA May Contribute to the Proper Stabilization of the 3′ Domain

**Figure 2:**
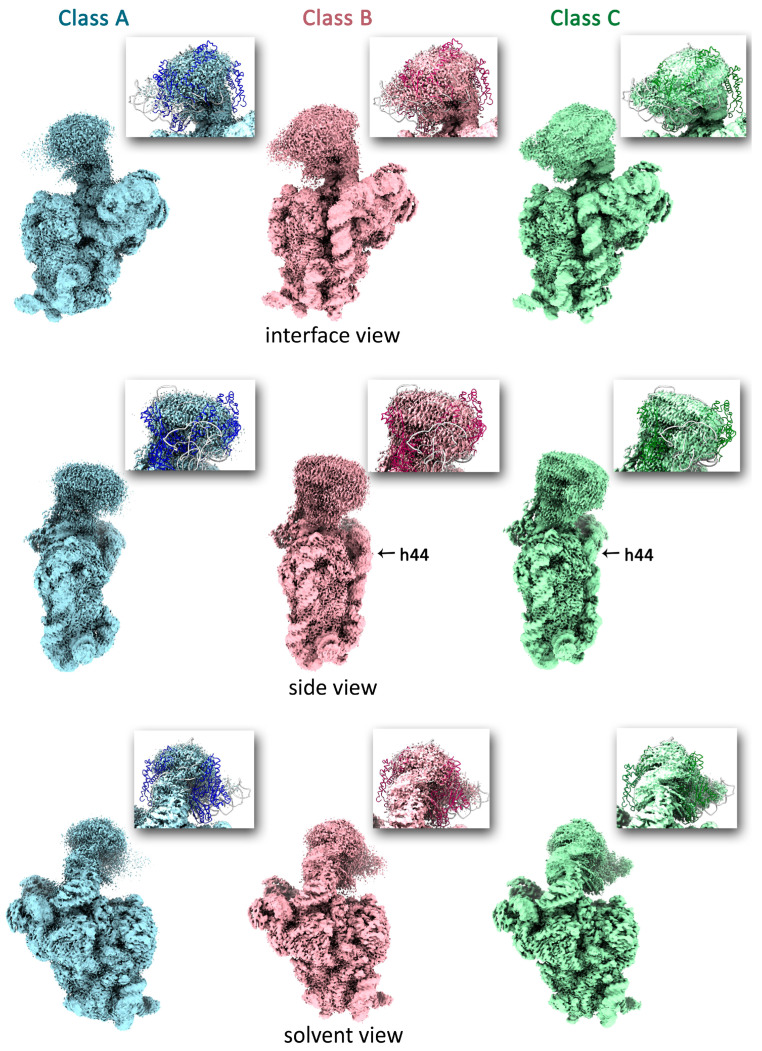
30S assembly intermediates in a Δ*lepA* strain. Cryo-EM maps of the three classes are displayed in interface, side, and solvent views. Particles are colored according to class: class A (blue, 22%), class B (pink, 20%), and class C (green, 57%). Insets show the atomic model of the wild-type *E. coli* 30S subunit head domain (PDB ID: 7AFD) fitted into the corresponding cryo-EM density maps from the Δ*lepA* 30S particles. This comparison highlights regions of the head domain of each class where density is absent. The 16S rRNA is shown as a light gray cartoon. Ribosomal proteins of the head domain are depicted as cartoons and colored blue, pink, and green for classes A, B, and C, respectively.

The structures of the immature 30S subunits purified from Δ*lepA* cells were determined by single particle cryo-EM coupled with image classification and three-dimensional (3D) reconstruction method. Initial rounds of 3D classification without a mask revealed continuous structural heterogeneity but did not yield well-separated classes. To resolve this heterogeneity and separate distinct conformational states, we performed a focused 3D classification using a mask encompassing the key helix 44 of 16S rRNA at the decoding center. This approach successfully separated the ∆*lepA* 30S particles into three discrete classes, namely A, B, and C, covering 99% of the particle population (Figure 2). Classes A, B, and C represent 22%, 20%, and 57% of the particle population, respectively. High-resolution cryo-EM maps were obtained for each of these classes: 2.25 Å for class A, 2.27 Å for class B, and 2.12 Å for class C. The structure of mature 30S subunits purified from wild-type *E. coli* cells was used as a reference to evaluate structural defects in immature particles [22].

All three classes of ∆*lepA* 30S particles represent intermediates with well-defined body (5′ domain) and platform (central domain). The structural variations occur in the head (3′ major domain) and the 3′-minor domain consisting of helices 44 and 45. The latter helices are essential components of the central decoding center (CDR) which also includes h27, the neck helix 28, and the central pseudoknot (PK), consisting of helices 1 and 2. The cryo-EM analysis of class A intermediates (22%) revealed a complete absence of helices 44 and 45, and smeared densities at the head domain location. The map for head domain has the fragmented density for rRNA and complete missing densities for ribosomal proteins. These missing densities for r-proteins could result from their physical lack or from intrinsic flexibility. Both scenarios are probable consequences of ongoing maturation events in the head domain. Thus, class A particles are characterized by a poor resolved head and disordered helices 44 and 45 at the decoding center.

In contrast, Δ*lepA* 30S particles of class B (20%) have ordered helices 44 and 45 and a more defined head. The entire rRNA density and the fragmented density of S14 are visible, while the densities for other r-proteins are missing in the head domain. The cryo-EM analysis of class B intermediates revealed significant distortions in helices 44 and 45. The upper segment of h44 is displaced outward, protruding from the interface of the 30S subunit that is in contact with the 50S subunit. This displacement also leads to a shift in the position of h45 in the decoding center. These results demonstrate that the decoding center is still unformed, while helices 44 and 45 are ordered.

Particles of class C (57%) conform to near-mature assembly intermediates with well-defined body, platform, 3′-minor domain and less well resolved head. The map for head domain has fragmented densities for proteins S9, S13, S14, S19, and missing densities for S3, S7, and S10. The most notable in class C particles is correctly docked helix 44 to the surface of the 30S subunit. This helix extends from the bottom of the body to the lower part of the head. The upper segment of helix 44, which is responsible for tRNA binding, bends toward helix 45 and the 3′ terminus of the 16S rRNA. This part of h44 latches to the intersubunit surface of 30S subunit forming the functional decoding center. Thus, the structure of the Δ*lepA* pre-30S subunit (class C) is nearly identical to that of wild-type 30S mature subunits [22].

Comparative analysis of the Δ*lepA* pre-30S subunit classes highlighted conformational differences localized to the h28 region. The neck helix 28 is structural switch delineating the CDR transition into a mature state. In classes A and B, helix h28 adopts a non-native secondary structure wherein residues U921-C924 base-pair with the 16S 3′ end (U1531-A1543). This interaction sequesters the 3′ end in the mRNA entry channel, rendering it unavailable for translation. Herewith, the h28/h44 and h44/h45 linkers form a labile helix-like structure, called h44^a^, in class B particles. This labile element is absent in class A particles because the corresponding linkers are disordered. In near-mature class C particles, helix 28 attains its native secondary structure, where residues U921-C924 interact with U1393-A1396. This base-pairing leads to the dissolution of the h44^a^ and stabilizes the h28/h44 and h44/h45 linkers. Accompanying this h28 reorganization, the 3′ end of the 16S rRNA swaps to the mRNA exit channel, making it available for protein synthesis. Thus, Δ*lepA* 30S particles with unformed CDRs (classes A and B) are in an inactive state, while class C particles with mature CDRs adopt an active conformation, allowing them to be incorporated into the pool of translating ribosomes.

**Figure 3:**
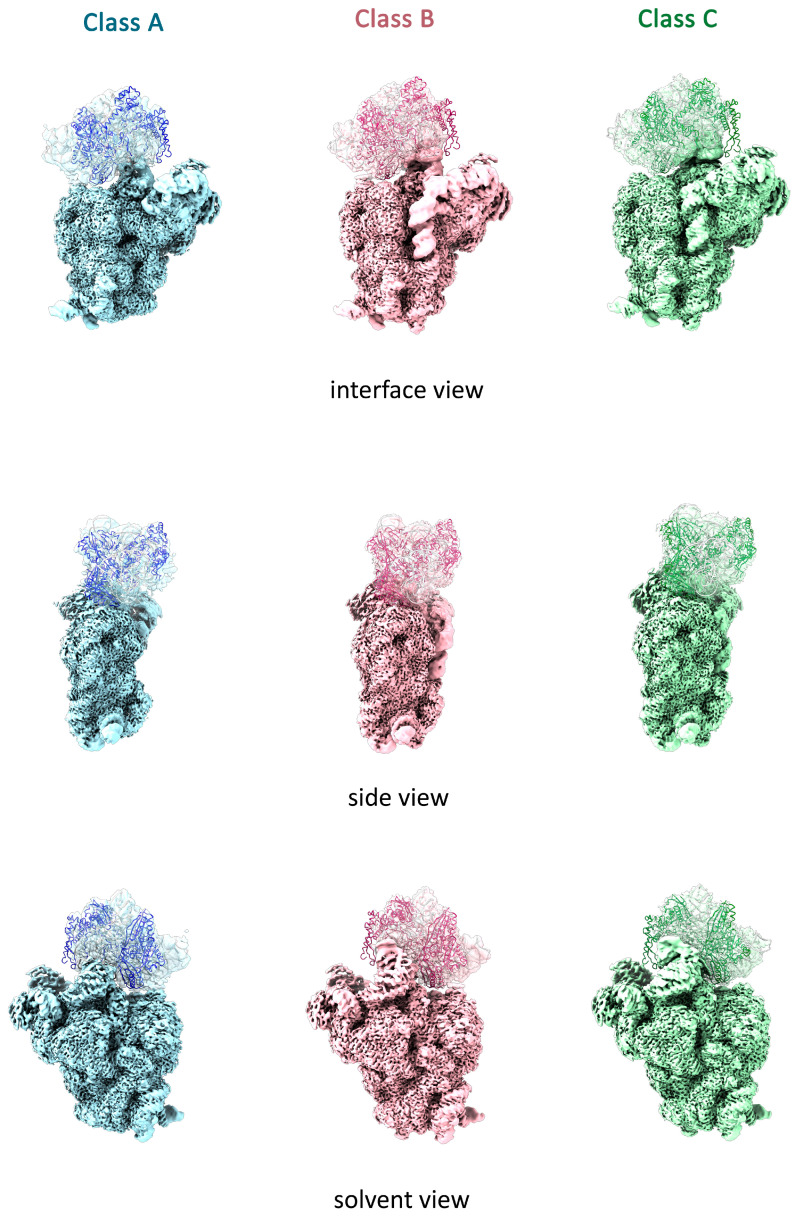
Cryo-EM maps of the 30S ribosomal head and body domains were obtained separately through signal subtraction of the corresponding regions followed by local refinement. The cryo-EM maps for the body domain are displayed as solid surfaces colored blue (class A), pink (class B), and green (class C). The corresponding head domain maps are shown as transparent surfaces in the same colors. The atomic model of the *E. coli* 30S subunit head domain (PDB ID: 7AFD) was fitted into the respective head maps. The 16S rRNA is depicted as a light gray cartoon. Ribosomal proteins of the head domain are shown as cartoons and colored blue, pink, and green for classes A, B, and C, respectively. For each class, interface, side, and solvent views are displayed. We observed a lack of densities for proteins S1 and S21 in maps from all classes. Proteins S1 and S21 are late-assembly r-proteins that interact weakly with the immature 30S subunit. Consequently, they may be lost during the preparation of cryo-EM samples. This interpretation is consistent with previous cryo-EM studies of immature 30S subunits from various assembly factor knock-out strains (e.g., Δ*rimM*, Δ*yjeQ*, Δ*era*, ∆*rsgA*∆*rbfA*), which also report the absence of density for S1 and S21 [20,23,24,25].

We performed local refinement separately for the head and body parts of each class of Δ*lepA* 30S particles after signal subtraction from the corresponding regions. The resulting local resolution estimates were as follows: class A intermediates achieved 2.24 Å (body) and 3.00 Å (head); class B, 2.26 Å and 2.66 Å; and class C, 2.10 Å and 2.34 Å, respectively (Figure 3). This approach significantly improved the resolution of the head domain density maps, enabling the visualization of all ribosomal proteins. The fragmented densities for protein S7 were observed, the complete densities for all other ribosomal proteins in the head domain became visible across all three particle classes. Consequently, the absence of these protein densities in the global maps and their subsequent appearance after local refinement indicate that the head domain possesses high intrinsic flexibility.

We identified specific post-transcriptional and post-translational modifications in Δ*lepA* 30S subunits, which function as essential checkpoints for ribosomal subunit maturation and are critical for ensuring accurate and efficient translation. The early stabilization of the central pseudoknot (PK) is evidenced by modifications in helix 28 (Ψ516, m^7^G527) and methylation of Asp89 in protein S12, both of which are present in all structural classes. Later stages of maturation are marked by the appearance of m^6^_2_A1518, m^6^_2_A1519, and m^7^G1516 modifications in class B and C particles. These modifications are crucial for stabilizing helix 45 and maintaining translational fidelity. The final maturation of the decoding center is indicated by the presence of m^4^Cm1402 and m^3^U1498 in helix 44, which are exclusive to class C particles. This chronological pattern of modifications mirrors the structural progression of the decoding center, confirming that these particles represent sequential intermediates on a productive assembly pathway.

We concluded that three structural classes (A, B, and C) of Δ*lepA* 30S particles represent productive late-stage assembly intermediates, serving as milestones along this pathway. The pathway progresses from class A particles, characterized by unformed helices h44/h45 and a poorly resolved head domain, through class B intermediates with a distorted decoding center and a more defined head, culminating in predominant near-mature class C subunits with a correctly docked h44 in the CDR, an accessible 3′ end of 16S rRNA for translation, and a flexible head. We interpret the progressive resolution of the head from class A to class C as a graduation from high flexibility to relative rigidity (Supplementary Materials Figure S1), suggesting that the correct docking of h44 and maturation of the decoding center are structurally linked to the stabilization of the head. Notably, even in near-mature class C particles, the head retains significant flexibility. This indicates that 30S subunit assembly in the absence of LepA can proceed to a functional state, yet the final stabilization of the head domain remains suboptimal. These findings are in good agreement with previous work, where Gibbs et al. used mass spectrometry to demonstrate that 3′ domain-binding proteins such as S3, S10 and S14 are underrepresented in immature 30S particles from Δ*lepA* strain, leading the authors to conclude that loss of LepA causes a specific defect in 30S subunit assembly [17]. Our structural data provides a mechanistic basis for this defect, revealing that LepA likely contributes to the final proper stabilization of 3′ domain, with a pronounced effect on the head of the 30S subunit.

## 3. Discussion

In 2017, Gibbs et al. demonstrated that cells lacking LepA accumulate immature 30S subunits [17]. They used fluorescence-based RNA electrophoresis to quantify the levels of 17S rRNA precursor, finding it to be 30–40% higher in the Δ*lepA* mutant than in the wild-type strain. Moreover, quantitative mass spectrometry revealed the underrepresentation of the late-assembly ribosomal proteins S3, S10, and S14—components of the 3′ domain—in ∆*lepA* 30S particles. Level of S3 was reduced by approximately 30%, while levels of S10 and S14 were 20%. Complementation with a plasmid-encoded LepA restored levels of these proteins to near stoichiometric amounts. Given that the ∆*lepA* mutation neither decreased the production rates of ribosomal proteins nor altered the 50S subunit composition, Gibbs and colleagues concluded that LepA plays a specific role in the late stage of 30S assembly, facilitating the incorporation of r-proteins necessary for the folding of 3′ domain.

Furthermore, the genetic studies have established a negative link between *lepA* and *rsgA*, wherein the ∆*lepA* mutation severely exacerbates the growth defect caused by ∆*rsgA* [14,17]. RsgA (YjeQ) is a ribosome-dependent GTPase and a known 30S assembly factor that is involved in the proper positioning of helix 44 in the decoding center [20,23,26]. The ∆*rsgA* 30S particles correspond to assembly intermediates that are also deficient in the r-proteins S2, S3, and S10 from the 3′ domain [17]. Despite the current lack of structural characterization of ∆*lepA* 30S particles, these findings support the idea that ∆*lepA* and ∆*rsgA* single mutants accumulate structurally similar ribosomal intermediates [17].

In this study, we isolated the immature 30S subunits from an *E. coli* strain lacking the *lepA* gene and characterized them by cryo-EM. The Δ*lepA* cells from the Keio collection accumulated unprocessed 17S rRNA-containing 30S ribosomal subunits. The levels of this 17S rRNA precursor were increased by 20–30% compared to the wild-type. Although this increase is less pronounced than that reported by Gibbs et al., our findings are in overall agreement [17]. The discrepancy is likely attributable to the different genotypic backgrounds of the bacterial strains used in our study and theirs. Despite confirmation by qPCR that the 17S rRNA precursor is unprocessed at both termini, we failed to detect significant densities for the extra nucleotides at the 5′ and 3′ ends of rRNA in our cryo-EM maps. This absence suggests a high degree of structural flexibility or disorder in these terminal sequences. This finding is similar to previous cryo-EM studies of immature 30S subunits [20,23,24], which also reported missing density for precursor-specific regions.

Our cryo-EM analysis revealed that immature 30S subunits from the Δ*lepA* strain represent in vivo assembly intermediates with a conserved architecture in the body and platform, but significant structural variations in 3′ domain (head and minor domain). This observation supports generally accepted assembly trajectories, in which the 5′ body domain forms first, followed by central platform and head with 3′ minor domain (including functionally essential helices 44 and h45 of decoding center) [27]. The structural heterogeneity of the 3′ domain in Δ*lepA* 30S intermediates is characterized by two parameters: the extent of maturation state of the 3′ minor domain and the scale of missing densities for ribosomal proteins in the head domain. The latter may reflect their physical absence, caused by ongoing assembly events in the head domain. However, our local refinement of the head domain revealed that densities for all r-proteins are present in all population of Δ*lepA* 30S particles. Therefore, the “missing densities” serve not as an indicator of incomplete assembly, but as a direct measure of the structural flexibility inherent to the head domain under these conditions.

Three structural classes (A, B, and C) of Δ*lepA* 30S particles represent discrete milestones along the late-stage 30S subunit assembly pathway. The pathway initiates with the least mature intermediate (class A), which demonstrates a highly flexible head domain and an unformed CDR due to the disordered helices h44 and h45. Progression to intermediates (class B) is marked by the formation of h44 and h55 in a distorted conformation, which precludes a functional decoding center. This transition coincides with partial stabilization of the head domain. The pathway ends in the predominant near-mature Δ*lepA* 30S particles (class C), where correct docking of h44 and h45 forms a functional decoding center. Although maturation of the decoding center is structurally linked to head domain stabilization, class C particles exhibit significant flexibility in the head domain. Our finding is consistent with the work of Gibbs et al., which showed that loss of LepA leads to the accumulation of immature 30S particles deficient in the tertiary head-domain proteins S3, S10, and S14 [17]. We therefore propose that LepA is essential for the 3′ head domain to adopt the proper conformation required for the correct incorporation of these ribosomal proteins. Given that 57% of Δ*lepA* 30S particles mature into a functional state but retain suboptimal head flexibility, we conclude that the final stabilization of the head domain is a distinct step that occurs post-assembly.

LepA is a ribosome-dependent GTPase that, like its paralogs EF-G and RF3 and related trGTPases such as TetM and BipA, is believed to function in the context of the 70S ribosome, as its GTPase activity is most strongly stimulated by 70S monosome [28]. Moreover, critical final maturation steps, including processing of the 16S rRNA 3′ terminus, are known to occur on the 70S ribosome, establishing a precedent for essential quality control events taking place after subunit association in bacteria [29]. In eukaryotes, a conceptually similar "test drive" mechanism exists, in which the GTPase eIF5B (a homolog of bacterial IF2) promotes the association of pre 40S and 60S subunits into a non translating 80S like complex [30]. This intermediate serves as a platform to verify critical functions including subunit joining, GTPase activation (eIF5B and Rli1), and A-site occupancy (by the Dom34 factor, a tRNA mimick) before final rRNA processing. Such a quality check ensures that only properly assembled subunits enter the translating pool.

Based on our structural observations and these established principles, we hypothesize that LepA acts at a late stage of 30S maturation within the context of a precursor 70S particle, where it likely contributes to the final stabilization of the 30S head domain. Elucidating the precise mechanism of LepA dependent quality control will require further investigation.

We compared our cryo-EM structures of Δ*lepA* 30S particles with previously determined structures of Δ*rsgA* 30S intermediates [20]. This analysis revealed that the Δ*rsgA* particles are structurally similar to the class B Δ*lepA* 30S intermediates. The class B state is characterized by the ordered formation of helices h44 and h45, wherein the upper segment of h44 remains displaced and fails to latch onto the 30S intersubunit surface. The structural similarity between the Δ*rsgA* and class B Δ*lepA* intermediates suggests that RsgA and LepA may have overlapping functions in 30S assembly. This functional redundancy might become important under stress conditions that impair RsgA activity. Notably, RsgA homologs are absent in eukaryotes and archaea. It is likely that LepA may be essential for the final accommodation of helix h44 into the decoding center in these domains of life. Together, we conclude that LepA may contribute to the proper stabilization of the 3′ domain of the 30S subunit during ribosome assembly.

## 4. Materials and Methods

### 4.1. E. coli Strains

*E. coli* K-12 derivative MG1655 (F-lambda-*ilvG*-*rfb-50 rph-1*) was used as a wild-type reference [31]. The ∆*lepA* strain JW2553 was obtained from the Keio collection [21].

### 4.2. Spot Assay

MG1655 and JW2553 (∆*lepA*) strains were grown in liquid LB medium at 37 °C to OD_600_ = 0.3–0.4 through an overnight culture and diluted to a series of concentrations, 10^0^, 10^−1^, 10^−2^, 10^−3^, 10^−4^, 10^−5^ and 10^−6^. Five microliters of each dilution was dropped to LB-agar plates and incubated at 17 °C, 37 °C and 42 °C overnight.

### 4.3. Ribosome Profile Analysis

MG1655 and JW2553 (∆*lepA*) strains were grown in 50 mL of liquid LB medium at 37 °C to OD_600_ = 0.4–0.5 through an overnight culture. Cultures were treated with 200 µg/mL chloramphenicol for 2 min by shaking to inhibit translation. Then, the cultures were chilled, harvested 5000 g for 25 min, resuspended in a 500 µL lysis buffer (20 mM Tris-HCl, pH 7.5, 10 mM MgCl_2_, 100 mM NH_4_Cl, 5 mM CaCl_2_, 0.1% NP-40, 0.4% Triton X-100 (*v*/*v*), 25 U/mL DNase Turbo (Invitrogen, Waltham, MA, USA), 1 mM chloramphenicol, 2 mg/mL lysozyme). Cell lysis was achieved by a single freeze–thaw cycle. Clarified lysates were loaded onto a 10–50% sucrose gradient (*m*/*v*), containing TAM buffer (20 mM Tris-HCl, pH 7.5, 100 mM NH_4_Cl, 10 mM MgCl_2_), and centrifuged at 35,000 rpm for 2.5 h in a SW41 rotor (Beckman Coulter, Brea, CA, USA). Gradients were analyzed using A_254_ absorption with a fractionation system.

### 4.4. Gel Electrophoresis Assay

The rRNAs from the purified ribosome subunits (prepared as described in [22]) were extracted by vortexing with equal volume of phenol and subsequent centrifugation at 10,000× *g*, 10 min. Part of water phase was mixed with loading dye solution (1× TBE buffer, 10% sucrose, 0.36% bromphenol blue and 0.36% xylene cyanol) and loaded on the gel composed of 0.7% agarose and 0.9% Synergel in 0.5× TBE buffer. rRNA was separated at 80 V, 3 h, and stained in 1 μg/mL ethidium bromide.

### 4.5. 17S rRNA Measurement with Quantitative PCR

Cells were cultivated in 60 mL of liquid LB medium at 37 °C to OD_600_ = 0.2, then collected by centrifugation, and lysed in 500 µL of lysis buffer 20 mM Tris-HCl, pH 7.5, 10 mM MgCl_2_, 100 mM NH_4_Cl, 5 mM CaCl_2_, 0.1% NP-40, 0.4% Triton X-100, 2 mg/mL lysozyme. RNA was isolated from a 100 µL aliquot using 400 µL of QIAzol Lysis Reagent (Qiagen, Germantown, MD, USA) and the Direct-zol RNA Miniprep Kit (Zymo Research, Irvine, CA, USA), following the manufacturer’s instructions. Next, 2 µg of total RNA was treated with dsDNase, and cDNA was synthesized using the Maxima H Minus First Strand cDNA Synthesis Kit (Thermo Fisher Scientific, Waltham, MA, USA).

Quantitative real-time PCR (qPCR) was performed on a DTlite Real-Time PCR System (DNA Technology, Risskov, Denmark) using qPCRmix-HS SYBR + LowROX reaction mixture (Evrogen, Moscow, Russia). A 25 µL aliquot of the reaction mixture contained 1/20 of the RT reaction mixture and 0.25 µM primers (see Table 1). The following cycling conditions were applied: an initial denaturation step at 95 °C for 2 min, followed by 30 cycles of denaturation at 95 °C for 15 s, annealing at 58 °C for 15 s, and extension at 72 °C for 20 s. The locations of the primers are indicated in Figure 1d.

The abundance of 17S amplicons was normalized to that of 16S + 17S as follows. For each 17S measurement, we calculated: (1) ΔCt as Ct^17S^ subtracting the mean of the technical replicates of Ct^16S+17S^; (2) ΔΔCt as ΔCt subtracting the mean of all (technical and biological) replicates of ΔCt in the WT strain (both for WT and *ΔlepA* strains); (3) 2^−ΔΔCt^ value. Then, for 2^−ΔΔCt^, we estimated the mean, the standard deviation, and the statistical significance (two-sided Student’s *t*-test of Δ*lepA* versus WT) across all technical and biological replicates. For each strain, RNA was extracted from three independent cultivations (biological replicates). For each cDNA sample, qPCR was performed in triplicate (technical replicates).

### 4.6. Isolation of Immature ΔlepA 30S Particles

Immature Δ*lepA* 30S subunits were isolated using a protocol previously established for Δ*rbfA* 30S subunits [22]. Briefly, Δ*lepA* cells were grown to an OD_600_ of ~0.2, and cell pellet was ground with alumina powder. The crude ribosomes were purified by three rounds of ultracentrifugation through a sucrose cushion buffer (20 mM Tris-HCl, pH 7.5, 500 mM NH_4_Cl, 10.5 mM MgCl_2_, 0.5 mM EDTA, 1.1 M sucrose, 3 mM 2-mercaptoethanol). The Δ*lepA* 30S particles were separated from 70S ribosomes by centrifugation through a 15–30% sucrose gradient in buffer (20 mM Tris-HCl, pH 7.5, 60 mM NH_4_Cl, 5 mM MgCl_2_, 3 mM 2-mercaptoethanol) for 12 h at 24,000 rpm using an SW28 rotor (Beckman). Fractions corresponding to free 30S subunits were collected, pelleted by ultracentrifugation, and resuspended in TAKM7 buffer (50 mM Tris-HCl, pH 7.5, 70 mM NH_4_Cl, 30 mM KCl, 7 mM MgCl_2_).

### 4.7. Cryo-Sample Preparation

For cryo-EM analysis, the Δ*lepA* 30S particles were purified by gel-filtration with a Superose^®^ 6 Increase 10/300 column (GE Healthcare, Uppsala, Sweden) equilibrated with HAKM_7_ buffer (50 mM HEPES-KOH, pH 7.5, 70 mM NH_4_Cl, 30 mM KCl, 7 mM MgCl_2_). The 30S-containing fractions were pooled and diluted to a concentration of 0.2 µM. A 3 µL of the sample with a 0.2 µM concentration was applied to the Quantifoil R1.2/1.3 300 mesh Cu grid coated with about 2 nm amorphous carbon layer (UTC), which was not glow-discharged prior to sample application. The grid was plunge-frozen in liquid ethane using a Vitrobot Mark IV (Thermo Fisher Scientific, USA) with the following settings: chamber humidity 100%; chamber temperature 4 °C; blotting time 3 s; blotting force 0. The grid was then stored in liquid nitrogen until use.

### 4.8. Cryo-EM Data Collection

Cryo-EM data were collected on a Titan Krios 60–300 transmission electron microscope (Thermo Fisher Scientific, USA) equipped with a field emission electron gun X-FEG (Thermo Fisher Scientific, USA), spherical-aberration corrector (CEOS GmbH, Heidelberg, Germany), a post-column BioQuantum energy filter (Gatan, Pleasanton, CA, USA) and a K3 direct electron detector (Gatan, USA) in standard counting (non-CDS) mode using SerialEM 4.055 [32] and 9-hole (2 exposures per hole, 18 exposure groups in total) image-shift data acquisition strategy at the National Research Centre “Kurchatov Institute”. The microscope was operated at 300 kV with a nominal magnification of 81,000×, corresponding to a pixel size of 0.863 Å at the specimen level, and an electron energy selecting slit of 20 eV. A total dose of 72 e^−^/Å^2^ within a 4 s exposure time was fractionated into 80 frames, resulting in an electron dose of 0.9 e^−^/Å^2^ per frame. A total of 7188 movies were collected in a nominal defocus range from −0.6 to −1.6 μm with a step of 0.1 μm. Detailed parameters of data acquisition are listed in Table S1.

### 4.9. Cryo-EM Data Processing

The initial cryo-EM data were pre-processed in Warp ver. 1.0.9 [33] global and local motion estimation, CTF model estimation, particle picking using a re-trained deep CNN BoxNet were performed for all collected movies. Data were inspected in a semiautomated manner using Warp’s thresholds for estimated defocus (less than 2.5 μm), resolution (less than 3 Å) and average motion per frame (less than 2 Å). A total of 2,313,522 particles were picked from 6786 selected images and extracted into 440 pixel boxes (Supplementary Materials Figure S2). The motion-corrected particle stacks were imported into CryoSPARC ver. 4.6.1 [34] for further data processing.

Several rounds of 2D classification were performed using 50 classes at each step to maximize the number of true-positive particles, remove 50S ribosomal particles, and other junk non-ribosomal particles. Reference-free 2D classification revealed accurate 30S particles with a clear secondary structure of the body domain and highly mobile head domain. The cleaned subset of 1,929,866 30S particles was used for ab initio initial map reconstruction and preliminary refinement. Following rounds of non-uniform refinement, per-particle defocus adjustment and per-exposure-group higher-order CTF terms adjustment resulted in a 2.12 Å resolution consensus map (in accordance with gold-standard FSC).

To prepare data for beam-induced motion correction using Bayesian polishing approach in RELION ver. 5.0.0 [35] csparc2star.py script from UCSF pyem collection [DOI: https://doi.org/10.5281/zenodo.3576630] and self-written bash script to parse particle coordinates from Warp’s outputs were used. Bayesian polishing was performed with the default motion parameters (σ_vel_ = 0.2 Å/(e^−^Å^−2^), σ_div_ = 5000 Å, σ_acc_ = 2 Å/(e^−^Å^−2^) without training step that improved consensus-map to 2.05 Å. The particle stack was split based on the bimodal per-particle scale factor distribution. Subsequent processing was applied to the particles corresponding to the high-scale peak. The resulting 1,339,738 particle set was subjected to further 3D classification in cryoSPARC. The classification was performed using a focused mask on helix h44 of the 30S ribosomal subunit, which yielded three major classes of *E. coli* 30S Δ*lepA* small ribosomal subunit: class A (297,347 particles), class B (269,839 particles) and class C (771,814 particles). For each of the resulting classes, non-uniform refinement was conducted. A final resolution was estimated at 2.25 Å, 2.27 Å and 2.12 Å for class A, class B and class C, respectively. Subsequently, signal subtraction was performed for the head and body domains, followed by local refinement for each region. The local resolutions for the body and head domains were 2.24 Å and 3.00 Å for class A, 2.26 Å and 2.66 Å for class B, and 2.10 Å and 2.34 Å for class C, respectively (Supplementary Materials Figure S3). All maps were locally filtered based on the local resolution estimates using the implementation in the cryoSPARC software package.

### 4.10. Model Building

PDB structures 7NAT, 7NAX and 7OE1 (cryo-EM reconstructions of *E. coli* 30S ribosomal subunit) were used as references for body domain modeling of *E. coli* 30S ∆*lepA* class A, class B and class C, respectively. Models were fitted into corresponding cryo-EM maps as a rigid body using UCSF Chimera (ver. 1.17.3) and ChimeraX (ver. 1.5). Protein and rRNA chains were visually inspected in WinCoot (ver. 0.9.8.93) and manually adjusted into the density map. The resulting models were built by several rounds of real-space refinement in the Phenix program suite and manual adjustments in WinCoot. Model validation was performed using the MolProbity tool (implemented in Phenix ver. 1.20.1-4487). Model-building parameters and refinement statistics are summarized in Supplementary Table S2.

## Figures and Tables

**Table 1:** Primers for qPCR.

Amplicon	Lenght, bp	Position (Relative to the 5′ End of Processed 16S rRNA)	Primer Name	Sequence
16S + 17S	100	6–27	Fw(16S rRNA)	GAAGAGTTTGATCATGGCTCAG
		87–105	Rw(16S rRNA)	CCACTCGTCAGCAAAGAAG
17S_5′	125	(−77)–(−98)	Fw(17S rRNA_5′)	ACGGATTCTTAACGTCGCAAG
		5–27	Rw(17S rRNA_5′)	CTGAGCCATGATCAAACTCTTCA
17S	142	(−11)–(−37)	Fw(17S rRNA)	TCATTACGAAGTTTAATTCTTTGAGCG
		87–105	Rw(16S rRNA)	CCACTCGTCAGCAAAGAAG
17S_3′	110	1456–1474	Fw(17S rRNA_3′)	AGGGCGCTTACCACTTTGT
		1538–1562	Rw(17S rRNA_3′)	CTGCAAAGTACGCTTCTTTAAGGTAAGG

## Data Availability

The cryo-EM maps have been deposited in the EMDataBank for 30S head, body, and consensus structures for class A, B and C with the following accession numbers: 67198, 67203, 67195, 67199, 67202, 67196, 67200, 67201, and 67197, respectively. The coordinates for the atomic models built for the 30S body domain for class A, B and C have been deposited in the Protein Data Bank (PDB codes ID 9XTE for class A; ID 9XTD for class B; ID 9XTC for class C).

## Supplementary Materials

The following supporting information can be downloaded at: https://www.mdpi.com/article/10.3390/ijms27010489/s1.
