# Supplementary material for: The Conserved GTPase LepA May Contribute to the Final Proper Stabilization of the 3′ Domain of the 30S Subunit During Ribosome Assembly
Source: Int J Mol Sci. 2026 Jan 3;27(1):489. doi: 10.3390/ijms27010489 (PMC12787274; doi:10.3390/ijms27010489)
Supplement: Supplementary file 1 [file ijms-27-00489-s001.zip › ijms-4034124-supplementary.pdf]

Supplementary Materials

The conserved GTPase LepA may contribute to the final proper stabilization of the 3' domain of the 30S subunit during ribosome assembly

Olesya Kravchenko, Elena Maksimova, Timur Baymukhametov, Irina Eliseeva, Elena Stolboushkina

This file includes:

I. Supplementary Figure S1, S2, S3 with legend

II. Supplementary Table S1, S2

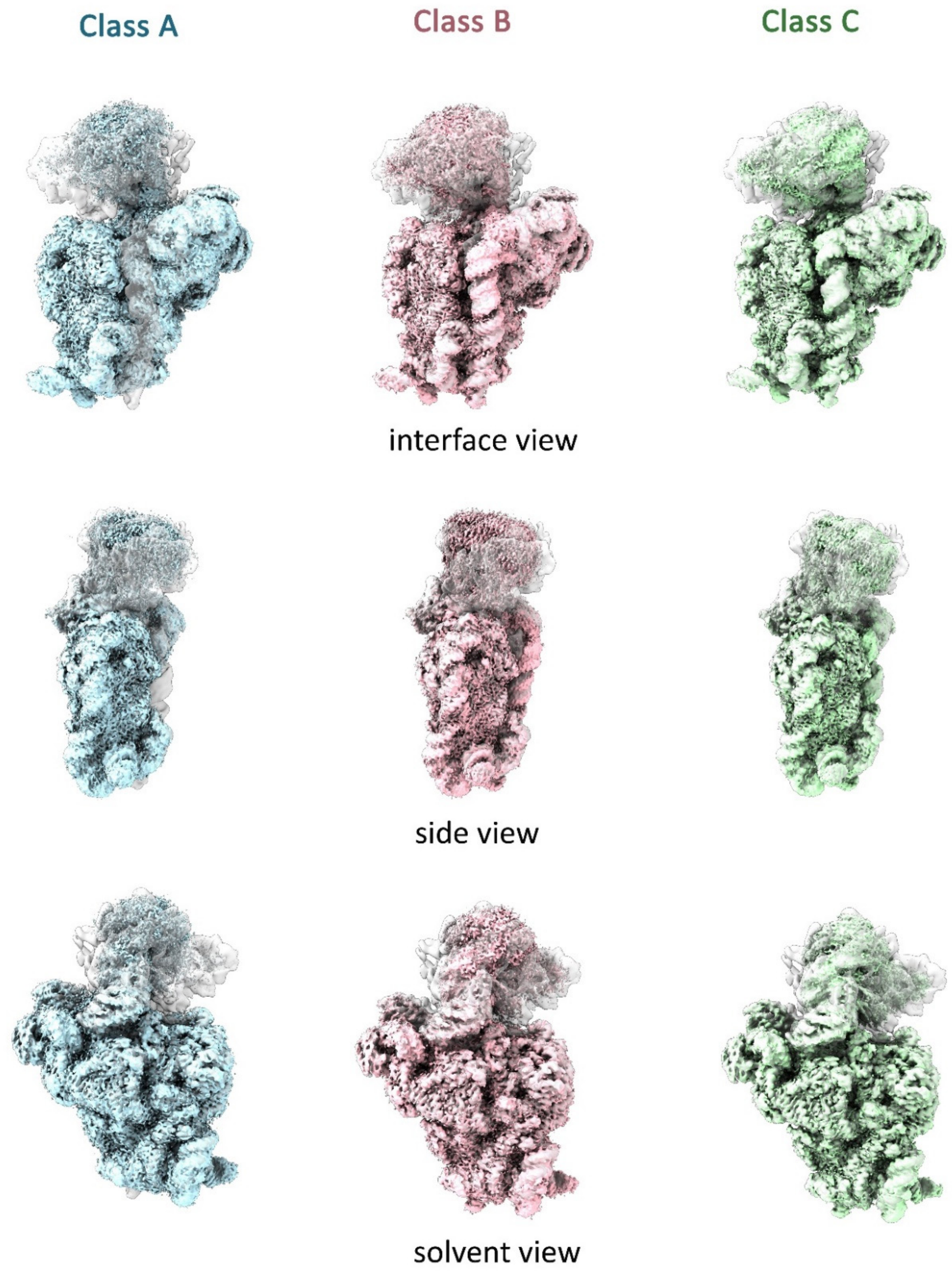

Supplementary Figure S1. Overlay of the cryo-EM density maps for the wild-type *E. coli* 30S ribosomal subunit (EMD-12857) and the *ΔlepA* 30S assembly intermediates. The *ΔlepA* 30S particles are displayed as solid surfaces, color-coded by class: class A (blue, 22%), class B (pink, 20%), and class C (green, 57%). The cryo-EM density of the wild-type 30S subunit is shown as a transparent light-gray surface. Interface, side, and solvent views are presented for each class.

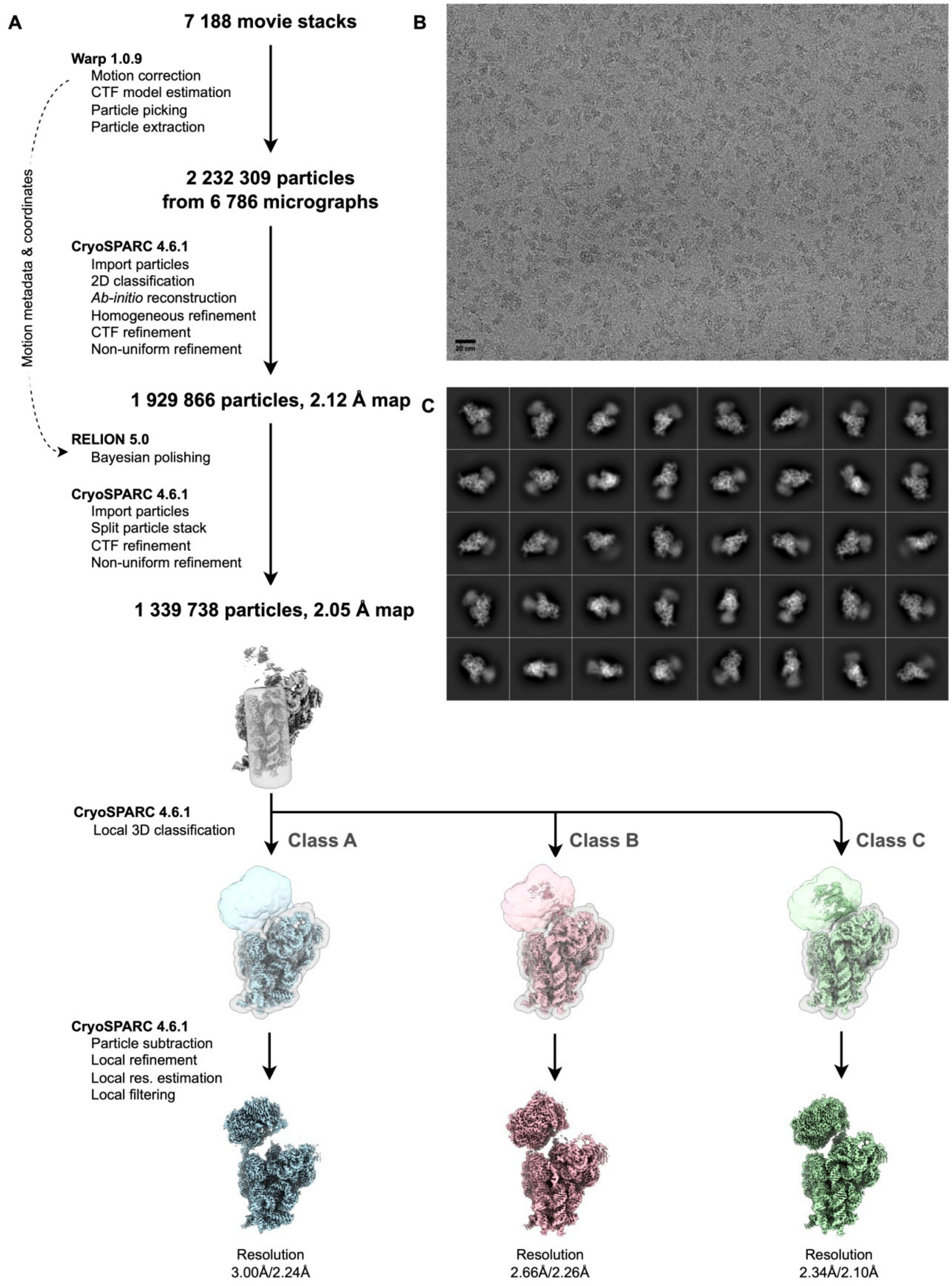

Supplementary Figure S2. Cryo-EM data processing workflow. (A) The workflow for the cryo-EM data processing. The cryo-EM consensus density map of 30S Δ*lepA* particles is shown in grey, and the mask used for the focused 3D classification is shown as a transparent grey cylinder. The three *E. coli* 30S Δ*lepA* classes are shown in blue (class A), pink (class B) and green (class C). Masks used for local refinement of the head domains are shown in the same colors as transparent surfaces. The masks used for the local refinement of the body domains are shown as

transparent grey surfaces. (B) A representative motion-corrected electron micrograph of 30S Δ*lepA* particles. The scale bar corresponds to 20 nm. (C) 2D classification of the cryo-EM data demonstrating different views of the particles.

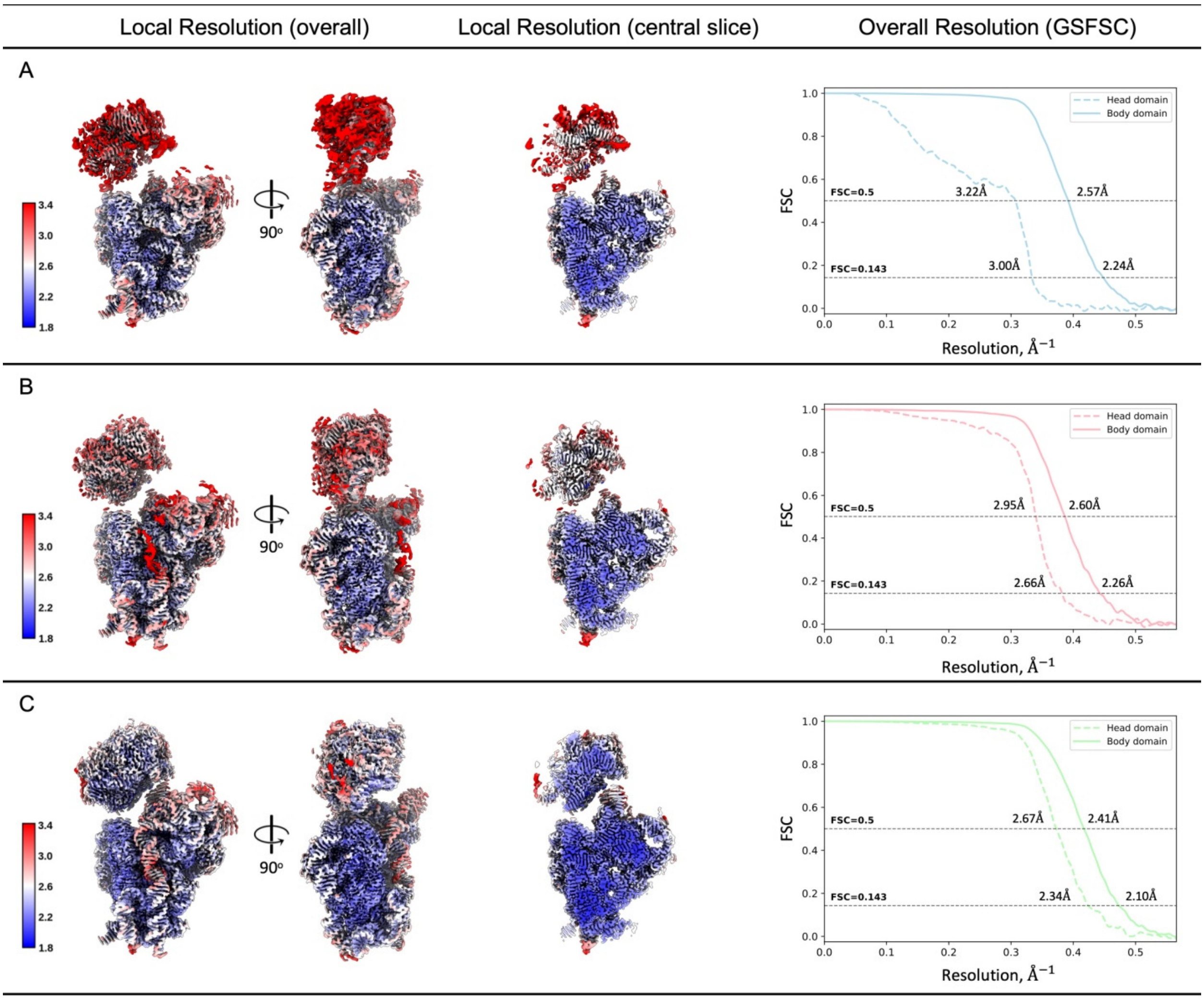

Supplementary Figure S3**.** Overall and local resolution estimation for the three major *E. coli* 30S Δ*lepA* classes. Left column: The composite cryo-EM density map of the 30S Δ*lepA* particles, with independently refined head and body domains. The maps are colored according to the local resolution, which was calculated using cryoSPARC. The color key is in Angstroms. Middle column: A slice through the 30S Δ*lepA* map, perpendicular to the intersubunit interface side view. Right column: Gold-standard FSC plots for the locally refined head and body domains. The global resolutions were estimated using with a gold-standard FSC (threshold at 0.143).

II. SUPPLEMENTARY TABLE

Table S1. Cryo-EM data collection and processing.

**Data collection**			
Software	SerialEM 4.055		
Grids	Quantifoil R1.2/1.3 300 mesh Cu UTC		
Microscope	Titan Krios		
Energy filter, Camera	BioQuantum, K3		
Voltage, kV	300		
Condenser apertures C1, C2, µm	2000, 100		
Spot size	5		
Illumination area, µm	1		
Energy selecting slit width, eV	20		
Nominal magnification	81 000x		
Calibrated pixel size, Å	0.863		
Nominal defocus range, µm	from -0.6 to -1.6		
Total exposure time, s	4		
Total electron dose, $e^-/Å^2$	72		
Number of frames per stack	80		
Electron dose per frame, $e^-/Å^2$	0.9		
Number of stacks (movies) collected	7 188		
**Data processing**			
Software	Warp 1.0.9, cryoSPARC 4.6.1, RELION 5.0		
Number of stacks (movies) used	6 786		
[1]Calibrated defocus range, median, 1st and 3rd quartile, µm	-0.9, -0.7, -1.2		
Initial number of particles	2 232 309		
Particle box size, px	440		
Final number of particles (consensus-map)	1 339 738		
[2]Final consensus-map $FSC_{0.143}$ resolution, Å	2.05		
[3]Final consensus-map map estimated B-factor, $Å^2$	60.2		
	Class A	Class B	Class C
Final number of particles per class	297 347	269 839	771 814
[2]Final $FSC_{0.143}$ resolution, Å (30S)	2.25	2.27	2.12
[3]Final map estimated B-factor, $Å^2$ (30S)	59.0	57.0	59.1
[2]Final $FSC_{0.143}$ resolution, Å (body domain)	2.24	2.26	2.10
[3]Final map estimated B-factor, $Å^2$ (body domain)	48.2	48.9	51.6
[2]Final $FSC_{0.143}$ resolution, Å (head domain)	3.00	2.66	2.34
[3]Final map estimated B-factor, $Å^2$ (head domain)	69.8	70.8	72.1
**Data availability**			
EMDB ID (30S)	67195	67196	67197
EMDB ID (body domain)	67203	67202	67201
EMDB ID (head domain)	67198	67199	67200

[1]Calibrated range and median of the average defocus values for 6 786 micrographs used, estimated using Warp;

[2]Resolution values after FSC-mask auto-tightening procedure estimated using CryoSPARC;

[3]B-factror value estimated based from last iteration Guinier plot using CryoSPARC;

Table S2. Refinement and validation statistics.

	Class A (body domain)	Class B (body domain)	Class C (body domain)
**Refinement**			
Initial model used	7NAT	7NAX	7OE1
Model resolution, Å	2.24	2.26	2.10
Non-hydrogen atoms	35512	37506	39859
Protein residues	1433	1462	1445
Nucliotedes	1026	1128	1176
Water	2231	1869	3243
Magnesium ion	34	39	39
B-factor, Å			
ptotein	70.72	57.10	46.92
RNA	109.82	104.16	81.97
r.m.s. deviation			
Bond length, Å	0.007	0.004	0.003
Bond angles, °	0.762	0.613	0.590
**Validation**			
MolProbity score	1.69	1.57	1.75
Clash score	8.77	7.95	10.35
Poor rotamer, %	0.00	0.00	0.00
Ramachandran plot			
Favored, %	96.59	97.26	96.61
Allowed, %	3.41	2.74	3.39
Disallowed, %	0.00	0.00	0.00
**Data availability**			
PDB ID	9XTE	9XTD	9XTC
EMDB ID	67203	67202	67201
